# Identifying the dominant transport mechanism in single nanoscale pores and 3D nanoporous media

**DOI:** 10.1016/j.fmre.2021.12.010

**Published:** 2022-01-05

**Authors:** Ying Yin, Zhiguo Qu, Maša Prodanović, Christopher J. Landry

**Affiliations:** a MOE Key Laboratory of Thermo-Fluid Science and Engineering, School of Energy and Power Engineering, Xi'an Jiaotong University, Xi'an 710049, China; b Hildebrand Department of Petroleum and Geosystems Engineering, The University of Texas at Austin, Austin, TX 78712, USA

**Keywords:** Porous media, Viscous flow, Mass diffusion, Apparent permeability, Apparent diffusivity, Lattice Boltzmann method

## Abstract

Gas transport mechanisms can be categorized into viscous flow and mass diffusion, both of which may coexist in a porous media with multiscale pore sizes. To determine the dominant transport mechanism and its contribution to gas transport capacity, the gas viscous flow and mass diffusion processes are analyzed in single nanoscale pores via a theoretical method, and are simulated in 3D nanoporous media via pore-scale lattice Boltzmann methods. The apparent permeability from the viscous flow and apparent diffusivity from the mass diffusion are estimated. A dimensionless parameter, i.e., the diffusion-flow ratio, is proposed to evaluate the dominant transport mechanism, which is a function of the apparent permeability, apparent diffusivity, bulk dynamic viscosity, and working pressure. The results show that the apparent permeability increases by approximately two orders of magnitude when the average Knudsen number (*Kn*_avg_) of the nanoporous media or Knudsen number (*Kn*) of single nanoscale pores increases from 0.1 to 10. Under the same conditions, the increment in the apparent diffusivity is only approximately one order of magnitude. When *Kn* < 0.01, the apparent permeability has a lower bound (i.e., absolute permeability). When *Kn* > 10, the apparent diffusivity has an upper bound (i.e., Knudsen diffusivity). The dominant transport mechanism in single nanoscale pores is the viscous flow for 0.01 < *Kn <* 100, where the maximum diffusion-flow ratio is less than one. In nanoporous media, the dominant transport relies heavily on *Kn*_avg_ and the structural parameters. For nanoporous media with the pore throat diameter of 3 nm, *Kn*_avg_ = 0.2 is the critical point, above which the mass diffusion is dominant; otherwise, the viscous flow is dominant. As *Kn*_avg_ increases to 3.4, the mass diffusion is overwhelming, with the maximum diffusion-flow ratio reaching ∼4.

## Highlights

• Mass diffusion and viscous flow processes are simulated via pore-scale lattice Boltzmann methods.
• Apparent permeability and diffusivity are predicted in single nanopores and nanoporous media.
• A dimensional diffusion-flow ratio is proposed to evaluate the dominant transport mechanism.
• Dominant transport mechanism is elucidated in single nanopores and nanoporous media.

## Graphical abstract

**Figure:**
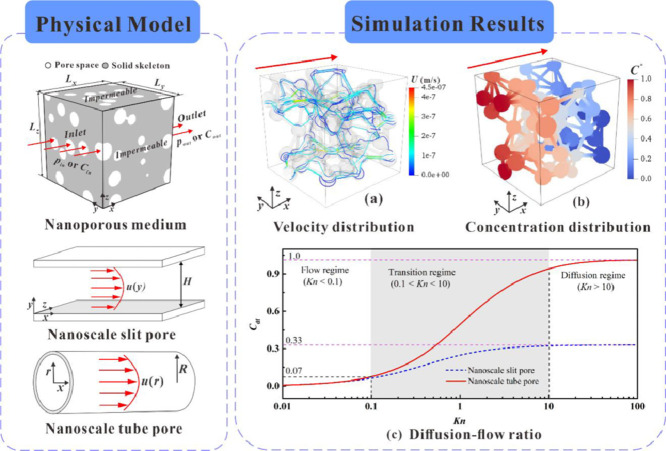

## 1 Introduction

Gas transport in porous media has gained much attention owing to its wide applications, such as optimizing the performance of fuel cells [1], enhancing the efficiency of catalytic reactions [2], and developing methane gas from unconventional reservoirs (e.g., shale gas [3] and coalbed methane [4]). Gas transport is a generalized concept that can be classified into viscous flow driven by a pressure gradient, and mass diffusion driven by a concentration gradient. Although the two are common forms of gas transport, their underlying mechanisms are quite different.

In general, the viscous flow is a directionally bulk motion of molecules driven by an external force such as a pressure gradient. Due to the fluid viscosity, the velocity profile over a cross section is nonuniform, whose feature is related to the pore shape, pore size, viscosity, and pressure gradient. In a porous media, the gas molecules prefer passing through pathways where the viscous drag (which is associated with the characteristic length, gas viscosity, and velocity) is the lowest. In a fracture-matrix system, for instance, the gas flow ability in the fracture is much higher than that in the tight matrix [5]. In contrast, mass diffusion is the gas molecules migration in a Brownian movement, showing no obvious preferences in the fracture and matrix, such that the fracture has a limited improvement of the gas diffusion ability [6]. Moreover, governing equations to describe the two transport behaviors are different [7]: the viscous flow is described by Navier–Stokes (N-S) equations, while the mass diffusion is described by Fick's law of mass diffusion [8]. As a result, a parabolic velocity profile for the viscous flow can be observed, while a plug concentration profile for the mass diffusion is observed when the gas passes through a simple pore in a steady state. The above distinctions thus suggest that the viscous flow and mass diffusion are different transport mechanisms. However, as the size of a simple pore decreases to the nanoscale under a low-pressure condition (where a continuum assumption is invalid and gas-solid interactions are strong), the shape of the velocity profile for the viscous flow transforms from a parabolic shape into a plug-like shape, similar to that of mass diffusion [9,10]. The transformation from viscous flow to mass diffusion at a low pressure was reported in [11,12]. Furthermore, the pressure gradient can be transformed into the concentration gradient by applying the gas equation of state (EOS), which serves as the driving force of gas molecule motion in the form of mass diffusion [13,14]. The above similarities and connections suggest that the conversion between the two transport behaviors occurs under certain conditions. The two transport mechanisms may also coexist if a porous medium has multiscale pore sizes that vary over a broad range. This raises the question of the scope of application of the two transport mechanisms.

The classic seepage theory considers that fluids percolating through porous rock at a low velocity (Reynolds number, *Re* < 1) can be simplified to a viscous flow with a negligible inertial force, obeying Darcy's law [15,16]. The fluid transport ability is characterized by the absolute permeability, whose value depends solely on the inherent properties of the rock [17,18]. In contrast, the macroscopic mass diffusion (Knudsen number, *Kn* < 0.1) theory considers the gas transport through porous materials as a method of molecular diffusion (or bulk diffusion), where the intermolecular collisions of gases dominate instead of gas-wall collisions [19,20]. The overall diffusion ability of gas molecules through porous media is termed as the effective diffusivity, whose value is dependent on pore geometric structures [19]. The absolute permeability and effective diffusivity are called transport coefficients, which are commonly used to measure the gas transport ability through a porous medium. However, when the pore size is reduced to the nanoscale, new phenomena or mechanisms arise, which further changes the transport coefficients.

With regard to gas molecule transport through tight nanoporous materials (e.g., organic matter in shales) under a small pressure gradient, new mechanisms induced by nanoscale pores and low pressure, such as the slip effect and Knudsen diffusion, may alter the original macroscopic gas migration pattern [3,21]. The measured permeability of gas transport at the nanoscale is usually larger than the absolute permeability, which is referred to as the apparent permeability [22]. The increase in apparent permeability is caused by the slip effect or the so-called Klinkenberg effect, in which a nonzero velocity adjacent to solid walls can be observed [23,24]. Because the slip effect is particularly important, a considerable number of models and methods have been developed to capture this effect over the past decades.

Based on the capillary nanotube model, Javadpour [25] studied the shale gas transport behavior at the nanoscale. The gas transport mechanisms consisted of two parts: the viscous flow coupled with the slip effect, and the pure Knudsen diffusion. The total gas flow rate was a linear superposition of the two transport mechanisms. The results show that the contribution of Knudsen diffusion to the total mass flux was appreciable at a low pressure and small pore size. Wang et al. [26] modified the Javadpour model [25] by considering several diffusion mechanisms (i.e., molecular, transition, and Knudsen diffusion) instead of pure Knudsen diffusion. The chosen diffusion mechanism in the model was determined by the magnitude of Knudsen number (*Kn*). Such analytical models have attracted much attention owing to their simplicity. However, a later study [27] found that the superposition of the viscous flow with the slip effect and pure Knudsen diffusion caused a double correction because the slip effect was already incorporated into the viscous flow term through the slip boundary; hence, the additional correction from the Knudsen diffusion was an overcorrection.

Another theory that considers the slip effect is the dusty gas model (DGM), in which the total mass flow rate is viewed as the linear sum of the viscous flow and Knudsen diffusion [28]. The similarity between the DGM andJavadpour models [25] is that they treat the total mass flux as the sum of the viscous flow and mass diffusion. Their differences lie in the fact that the DGM does not include the slip effect in the viscous flow term, but uses the Knudsen diffusion term to correct the slip effect. The DGM model was employed by Chen et al. [29] to estimate the apparent permeability of the shale matrix. In their work, the mass flow rate of methane gas by way of the viscous flow and Knudsen diffusion were computed through the standard lattice Boltzmann method (LBM). Considering that the DGM is an empirical treatment, it requires further validation when it comes to the nanoporous media.

Currently, direct simulation of the LBM combined with an improved slip boundary and effective relaxation time has made it possible to accurately mimic the gas viscous flow in complex nanoporous media [27,30]. A local effective viscosity LBM (LEV-LBM) with a multiple relaxation time collision operator has enabled the direct modeling of the mixed viscous-ballistic flow of methane gas in nanoporous organic matter, and the estimation of the apparent permeability [27]. Later, a regularized LBM was proposed to consider the gas slip flow in a reconstructed nanoporous shale matrix in a wide *Kn* range [31,32]. The above studies (where the viscous flow is assumed to be the dominant transport) are significant when considering the slip effect of gas viscous flow in complex nanoporous materials.

Gas transport mechanisms also rely on the working conditions and structure of porous media; this means that mass diffusion can be noticeable under certain conditions [33]. Previous experimental measurements [34] indicated that the dominant transport mechanism for methane gas through a rock matrix saturated with nanoscale pores is more likely to be mass diffusion rather than viscous flow. When the gas diffuses in nanoporous media, multiple diffusion mechanisms (i.e., molecular, transition, and Knudsen diffusion) coexist in the void pore spaces owing to a broad pore size distribution [35]; the gas diffusion capability is termed as the apparent diffusivity. To capture the multiple gas diffusion behaviors, Yin et al. [36] proposed a local *Kn* concept and the local diffusivity LBM (LD-LBM) to study the methane gas diffusion in two-dimensional (2D) nanoporous organic matter. They found that the methane gas diffusion mechanism transformed from the Knudsen diffusion to molecular diffusion with the increase in pressure. Qu et al. [37] extended the 2D LD-LBM by considering the influence of the adsorbed gas and surface diffusion. They found that the pore-throat diameter is more important than the pore-body diameter in determining the gas diffusivity in nanoporous organic matter. These studies are crucial for exploring the mass diffusion mechanism at the nanoscale; however, they do not discuss the relationship between mass diffusion and viscous flow.

In summary, the above literature review showed that both viscous flow and mass diffusion have been widely studied. However, which transport mechanism is dominant in practical applications; what is the relationship between viscous flow and mass diffusion; and when will the conversion between the two mechanisms occur (especially in complex nanoporous media); these questions are still unclear. In this study, the gas viscous flow and mass diffusion in single nanopores are first analyzed. Then, the transport processes in a set of reconstructed three-dimensional (3D) nanoporous media are simulated through pore-scale LBMs, in which the viscous flow is mimicked by the LEV-LBM considering the slip effect, and the mass diffusion is mimicked by the LD-LBM considering multiple diffusion effect. To determine the dominant transport mechanism, a dimensionless parameter, i.e., the diffusion-flow ratio, is defined and expressed as a function of the apparent permeability, apparent diffusivity, bulk dynamic viscosity, and average pressure. The factors influencing the apparent permeability and apparent diffusivity (e.g., *Kn, Kn*_avg_, and geometric and topologic features of porous materials) are discussed. Finally, the relationship between the two transport mechanisms and the dominant transport mechanism are elucidated.

## 2 Analyzing of viscous flow and mass diffusion

To evaluate the relative magnitude between the viscous flow and mass diffusion when gas is transported through a medium, the direct approach is to separately calculate the gas mass flow rates under the same working conditions, and perform a comparison. In this method, a dimensionless number, i.e., the diffusion-flow ratio ($C_{\text{df}}$), is defined to determine the dominant transport mechanism. The diffusion-flow ratio ($C_{\text{df}}$) is the mass flow rate ratio of the mass diffusion ($M_{\text{diff}}$) and viscous flow ($M_{\text{flow}}$). It is apparent that $C_{\text{df}}=1$ serves as a critical point: when $C_{\text{df}}<1$, viscous flow is dominant; when $C_{\text{df}}>1$, mass diffusion is dominant. At the critical point, the mass flow rate calculated by the viscous flow is equal to that calculated by the mass diffusion. The further away from the critical point, the corresponding transport mechanism contributing to the transport capacity becomes more prominent. The value of critical point is independent of the pore morphology, but the value of $C_{\text{df}}$ does. The derivations of $M_{\text{flow}}$, $M_{\text{diff}}$, and $C_{\text{df}}$ for gas transport through a medium are presented as follows.

Because the gas velocity at the nanoscale is fairly low, the mass flow rate of the viscous flow ($M_{\text{flow}}$), either in single nanopores or nanoporous media, can be estimated via a formula in the form of Darcy's law (Eq. 1), in which the influence of the slip effect is incorporated into the apparent permeability term:

(1) $$M_{\text{flow}}=-\rho A\frac{k_{\text{app}}}{\mu_{0}}\frac{dp}{dx}.$$

where ρ is the gas density, A is the cross-sectional area of the host medium, $k_{\text{app}}$ is the apparent permeability, $\mu_{0}$ is the gas bulk dynamic viscosity, and dp/dxis the pressure gradient along the flow direction (*x*-direction).

Likewise, the mass flow rate of the mass diffusion ($M_{\text{diff}}$) can be estimated in the form of Fick's first law (Eq. 2), in which the influence of the multiple diffusion effect is incorporated into the apparent diffusivity term:

(2) $$M_{\text{diff}}=-AD_{\text{app}}M\frac{dC}{dx}$$

where M is the molar mass, $D_{\text{app}}$ is the apparent diffusivity, and dC/dx is the concentration gradient along the diffusion direction (*x*-direction). Since the single component gas is involved in the present work, the apparent diffusivity here belongs to the self-diffusivity.

Because the gas concentration is proportional to the pressure, the concentration gradient can be converted into a pressure gradient. The relationship between the gas concentration and pressure can be described by the real gas EOS as:

(3) $$Z=\frac{p}{CR_{\text{ig}}T}$$

where *Z* is the compressibility factor, *p* is the average pressure within the medium, and $R_{\text{ig}}$ is the ideal gas constant ($R_{\text{ig}}=8.314J\cdot K^{-1}\text{mo}l^{-1}$). The concentration gradient in Eq. 2 can be replaced by the pressure gradient by taking the derivative ofthe pressure with respect to the distance in Eq. 3 and substituting it into Eq. 2. The mass flow rate of the mass diffusion ($M_{\text{diff}}$) in the pressure gradient is given by:

(4) $$M_{\text{diff}}=-AD_{\text{app}}\frac{\rho }{p}\frac{dp}{dx}.$$

Note that the apparent permeability in Eq. 1 and apparent diffusivity in Eq. 4 should be computed according to the same medium, pressure, and temperature.

With the obtained expressions for $M_{\text{flow}}$ and $M_{\text{diff}}$, the relative magnitude of mass flow rates in the form of viscous flow and mass diffusion can be estimated using the previously defined dimensionless parameter, i.e., the diffusion-flow ratio ($C_{\text{df}}$) expressed as:

(5) $$C_{\text{df}}=\frac{M_{\text{diff}}}{M_{\text{flow}}}.$$

The expressions of $M_{\text{flow}}$ (Eq. 1) and $M_{\text{diff}}$ (Eq. 4) share some common parameters (i.e., ρ, A, and dp/dx), which are identical when the processes of viscous flow and mass diffusion evolve under the same conditions. Thus, substituting the expressions of $M_{\text{flow}}$ and $M_{\text{diff}}$ into Eq. 5 and removing the common parameters will yield a more tangible $C_{\text{df}}$ expressed as:

(6) $$C_{\text{df}}=\frac{D_{\text{app}}}{k_{\text{app}}}\frac{\mu_{0}}{p}.$$

In Eq. 6, $C_{\text{df}}$ is a general expression that depends only on the apparent diffusivity ($D_{\text{app}}$), apparent permeability ($k_{\text{app}}$), bulk dynamic viscosity ($\mu_{0}$), and average pressure (p), such that the relative magnitude of the mass flow rates of viscous flow and mass diffusion can be estimated through Eq. 6, as long as all the foregoing parameters are known. In the present work, Eq. 6 is applied to simple nanoscale pores (i.e., nanoscale slit and tube pores) and nanoporous media without loss of generality. The procedures for computing the diffusion-flow ratio of single nanoscale pores and nanoporous media are detailed in Sections 3 and 4, respectively.

## 3 Gas viscous flow and mass diffusion in the single nanopores

**Fig. 1:**
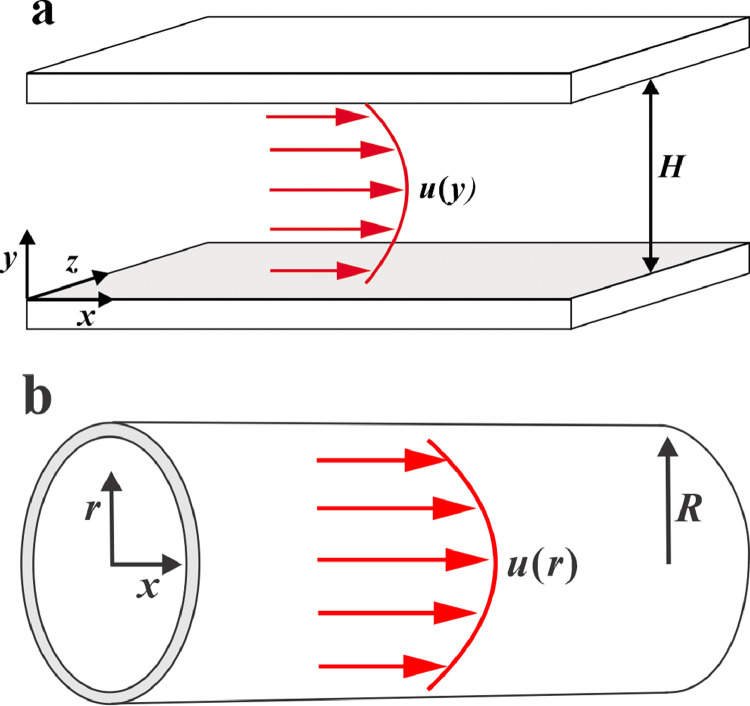
**Schematic of the gas flow in nanoscale slit and tube pores**. (a) Nanoscale slit pore. (b) Nanoscale tube pore.

Gas transport in single nanoscale pores are first examined owing to their popularity; more importantly, their analytical solutions are available under certain conditions. To derive the analytical solutions, it was assumed that the gas flow was under the fully developed condition (i.e., the gas velocity distribution varies in the transverse direction and is independent of the flow direction), obeying Poiseuille's law. Fig. 1a,b shows a schematic of the gas flow in the slit and tube pores, where *H* and *R* denote the height of the slit and the radius of the tube, respectively. Because of the existence of a viscous drag force, the parabolic velocity profiles can be observed along the transverse direction.

### 3.1 Apparent permeability for the slit and tube pores

The slip effect is evident when the gas flows at the nanoscale. To capture the slip effect, Beskok and Kariadakis [38] proposed the B-K model to describe the gas flow behaviors over the entire *Kn* range [39]. The B-K model also serves as a benchmark solution for validation of other models for owing to its accuracy [27,29]. To determine the apparent permeability ($k_{\text{app}}$) of the nanoscale slit and tube pores, the B-K model is also adopted in this study. The permeability of the nanoscale slit pore is given by:

(7) $$k_{\text{app}}=\frac{H^{2}}{12}\left(1+\frac{6Kn}{1+Kn}\right)\left(1+2.2Kn\right),$$

where the *Kn* is calculated by:

(8) $$Kn=\frac{\lambda_{0}}{H}.$$

Theunbound mean free path ($\lambda_{0}$) is calculated by:

(9) $$\lambda_{0}=\frac{1}{\sqrt{2}\pi nd_{m}^{2}},$$

where $d_{m}$ is the molecular diameter of the gas. The molecular diameter of methane gas can be calculated by the formula proposed by Speedy et al. [40]. n is the number density of gas molecules, which is obtained by solving the Peng–Robinson EOS [41]. The detailed procedures to calculate $d_{m}$ and n have been well documented in the previous study [6].

For the gas flow in the nanoscale tube, the apparent permeability in the B-K model is calculated by:

(10) $$k_{\text{app}}=\frac{R^{2}}{8}\left(1+\frac{4Kn}{1+Kn}\right)\left(1+\alpha Kn\right),$$

where the *Kn* is calculated by:

(11) $$Kn=\frac{\lambda_{0}}{R}.$$

In Eq. 10, *α* is an empirical parameter determined by the *Kn* and pore morphology. The calculation formula of *α* in a tube pore is asfollows [42]:

(12) $$\alpha =\frac{1.358}{1+0.1780Kn^{-0.4348}}.$$

Substituting Eq. 12 into Eq. 10, the apparent permeability of the nanoscale tube is written as:

(13) $$k_{\text{app}}=\frac{R^{2}}{8}\left(1+\frac{4Kn}{1+Kn}\right)\left(1+\frac{1.358Kn}{1+0.1780Kn^{-0.4348}}\right).$$

### 3.2 Apparent diffusivity for the slit and tube pores

The multiple diffusion effect is evident when the gas diffuses in nanoscale pores. To capture the multiple diffusion effect, Guo et al. [35] modified the single gas molecular diffusivity ($D_{M}$) by incorporating the *Kn*. The modified diffusivity is the apparent diffusivity ($D_{\text{app}}$) that can be estimated by Eqs. 14 and 15 for the nanoscale slit and tube, respectively:

(14) $$D_{\text{app}}=\frac{D_{M}}{1+1.8Kn},$$

(15) $$D_{\text{app}}=\frac{D_{M}}{1+0.9Kn}.$$

In Eqs. 14 and 15, the molecular diffusivity ($D_{M}$) is given by:

(16) $$D_{M}=\frac{3\pi }{16}\lambda_{0}\bar{v}\approx \frac{3}{5}\lambda_{0}\bar{v},$$

where $\bar{v}$ is the mean molecular velocity expressed as:

(17) $$\bar{v}=\sqrt{\frac{8k_{B}T}{\pi m}}=\sqrt{\frac{8R_{\text{ig}}T}{\pi M}}.$$

$D_{\text{app}}$ and $k_{\text{app}}$ are known for the single nanoscale slit and tube pores through the process described above. To obtain $C_{\text{df}}$, the values of $\mu_{0}$ and p need to be known; both are state parameters that depend on the temperature, pressure, and gas type.

### 3.3 Diffusion-flow ratio for the single nanopore

According to the gas kinetic theory [43,44], the relationship between the bulk dynamic viscosity and pressure can be expressed as:

(18) $$\frac{\mu_{0}}{p}=\lambda_{0}\sqrt{\frac{2M}{\pi R_{\text{ig}}T}}.$$

After determining the relationship between $\mu_{0}$ and p, the diffusion-flow ratio can be easily obtained. Substituting Eqs. 7, 14 and 18 into Eq. 6 yields the following expression for the diffusion-flow ratio in the nanoscale slit pore:

(19) $$C_{\text{df}}=\frac{144Kn^{2}}{\pi \left(5+9Kn\right)\left(1+\frac{6Kn}{1+Kn}\right)\left(1+2.2Kn\right)}.$$

Likewise, substituting Eqs. 13, 15 and 19 into Eq. 6 yields the following expression for the diffusion-flow ratio in the nanoscale tube pore:

(20) $$C_{\text{df}}=\frac{96}{5\pi }\frac{Kn^{2}}{\left(1+0.9Kn\right)\left(1+\frac{4Kn}{1+Kn}\right)\left(1+\frac{1.358Kn}{1+0.1780Kn^{-0.4348}}\right)}.$$

It should be noted that Eqs. 19 and 20 are suitable for the high *Kn* condition (*Kn* > 0.01) because the expressions of $D_{\text{app}}$ and $\mu_{0}/p$ are designed for the low-pressure condition. In the next section, the real gas effect is considered when calculating $D_{\text{app}}$ and $\mu_{0}/p$ in nanoporous media, so that $C_{\text{df}}$ is suitable for the entire *Kn* range.

## 4 Gas viscous flow and mass diffusion in nanoporous media

### 4.1 Physical model

**Fig. 2:**
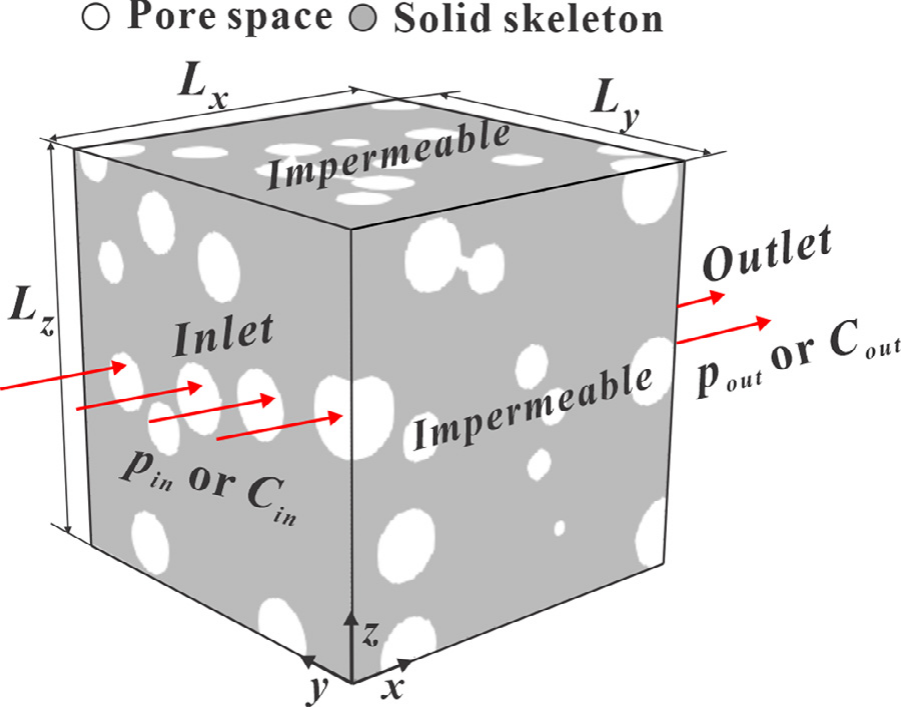
**Physical model for the gas transport in a porous medium**.

The physical model with respect to the gas transport in a porous medium is displayed in Fig. 2. The computational domain is a cubic box ($L_{x}=L_{y}=L_{z}=100\text{nm}$), where $L_{x}$, $L_{y}$, and $L_{z}$ denote the lengths in the *x*-, *y*-, and *z*-directions, respectively. The solid skeletons and void spaces are labeled in gray and white, respectively. Because the gas transport ability in the solid skeleton is negligible compared with that in void spaces, the solid skeletons are assumed to be impermeable, and the void pores are only transport spaces. The pressures at the inlet and outlet are fixed to produce a pressure gradient (0.1 MPa/m) along the flow direction (i.e., the *x-*direction). The average pore pressure is the arithmetic mean of the inlet and outlet pressures. The other four lateral boundaries are treated as impermeable solid walls. Because the slip effect is significant at the gas-solid interfaces when the pore size is at the nanoscale, N-S equations with a slip boundary condition and a local effective viscosity are solved to capture the slip effect. For the gas diffusion, Fick's second law with a local diffusivity is used to consider the multiple diffusion effect induced by the nanoscale pores. Because the analytical solutions of $D_{\text{app}}$ and $k_{\text{app}}$ are difficult to obtain in the nanoporous media, direct simulations of the LBM are conducted.

### 4.2 Reconstruction and characterization of nanoporous media

Because the most porous media can be simplified to pore-throat porous structure, this kind of porous media that consist of pore-throat units are generated for the flow and diffusion simulations. The reconstruction includes two steps: (1) randomly placing void spheres (i.e., pore bodies) into a solid cubic box until their volume fraction reach the set value ($\phi_{\text{pb}}$); and (2) connecting the void spheres to their nearest neighboring spheres by the void cylinder tubes (i.e., pore throats). The number of target neighboring spheres is determined by the coordination number (*N* = 5) [36,37]. The diameters of the pore bodies follow a normal distribution, with the mean value and standard deviation denoted by $d_{\text{pb},\text{avg}}$ and σ, respectively; the diameter of the pore throat is a constant denoted by $d_{\text{pt}}$. More information regarding the reconstruction of pore-throat porous media can be found in the literature [36].

**Fig. 3:**
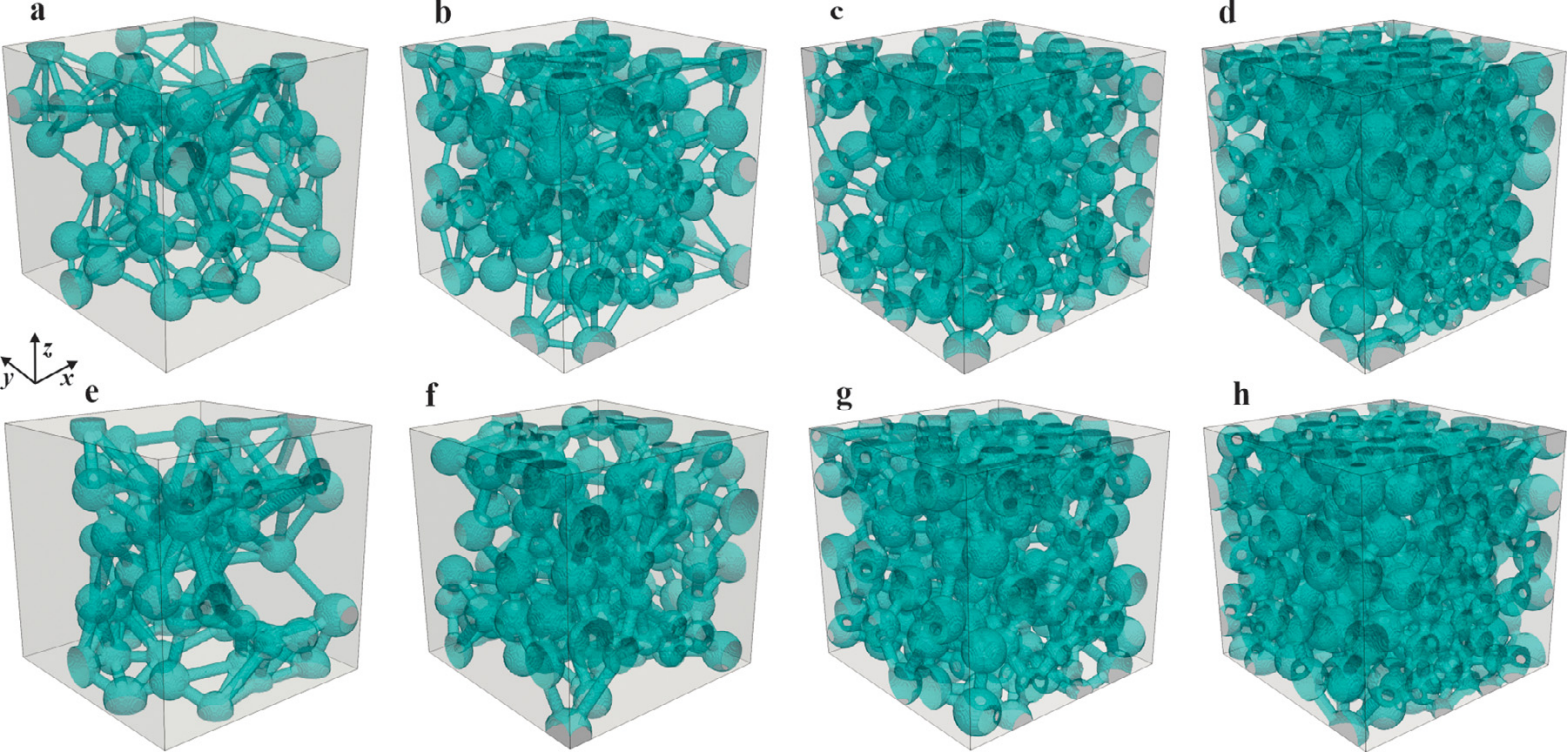
**Reconstructed pore-throat porous media**. (Detailed reconstruction parameters are listed in Table 1.)

A set of reconstructions with varying structural parametersare shown in Fig. 3. The void pore spaces and solid skeletons are shown as semitransparent green and gray, respectively. The size of the reconstructed domain is $N_{x}\times N_{y}\times N_{z}=200\times 200\times 200$ in a lattice unit. The lattice resolution is 0.5 nm per lattice. The average pore body diameter was fixed at 15 nm for all reconstructions, whereas the throat diameter changes per row. The throat diameters in the first and second rows are 3 nm (Fig. 3a–d) and 5 nm (Fig. 3e–h), respectively. The porosities of reconstructions in each row gradually increase from left to right (0.113–0.404 in the first row; 0.140–0.428 in the second row). In each column, the volume fraction of the pore body for the reconstruction is the same; hence, the reconstruction with the larger throat diameter has the higher volume fraction of pore throat and porosity (e.g., Fig. 3a,e). Table 1 summarizes the detailed reconstruction parameters for the pore-throat porous media shown in Fig. 3. In addition, it is noted that although the pore-throat structure shown in Fig. 3 looks similar to the previously reported pore network model (PNM) [45], but the calculation method and principle are totally different from the PNM. In fact, LBM mimics a real transport behavior, which can consider the converging and diverging information in the pore-throat porous media. However, PNM implements an oversimplification in the calculation, which makes it fail to consider this structure information. A detailed comparison can be found in Ref. [46].

**Table 1:** **Reconstruction parameters for the pore-throat porous media**.

Fig. 3	ε	$d_{\text{pt}}$ (nm)	$\phi_{\text{pb}}$	$d_{\text{pb},\text{avg}}$ (nm)	σ (nm)
a	0.113	3.0	0.1	15.0	1.5
b	0.214		0.2		
c	0.311		0.3		
d	0.404		0.4		
e	0.140	5.0	0.1	15.0	1.5
f	0.240		0.2		
g	0.340		0.3		
h	0.428		0.4		

**Fig. 4:**
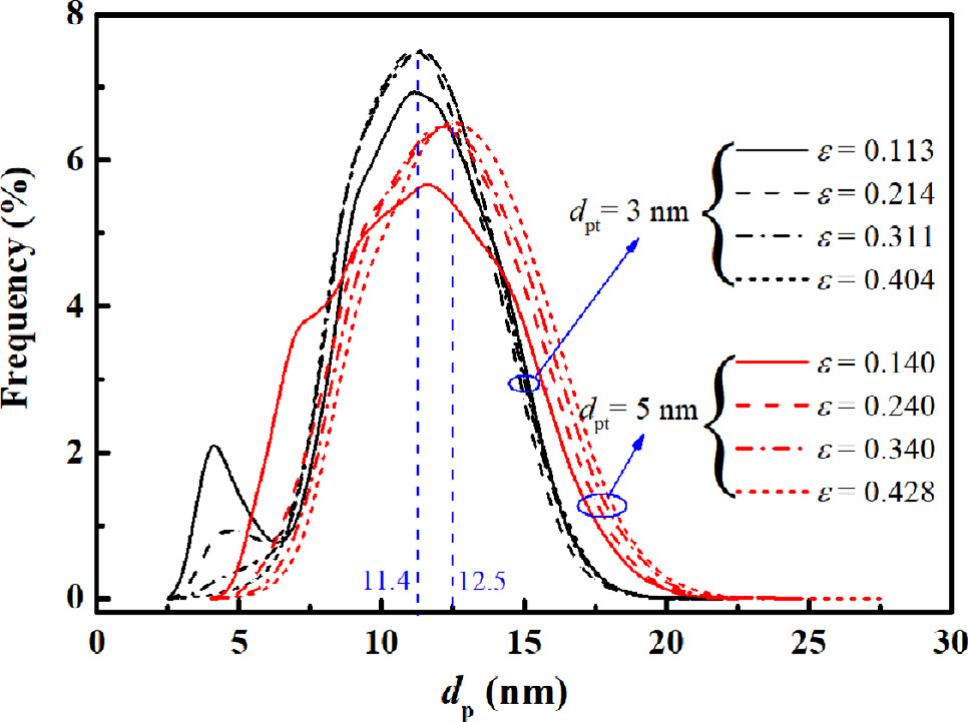
**Pore size distribution of pore-throat nanoporous media**.

The pore size distribution is a crucial parameter in measuring the varying features of the local pore size in a porous medium. To characterize the local pore size distributions in the reconstructed pore-throat porous media, a modified 13-direction average method [47] is adopted, which has been widely applied in the pore size characterization of porous media [29,36,37]. Fig. 4 shows the pore size distributions of the reconstructed pore-throat nanoporous media. Overall, the pore size distributions exhibited normal distribution, and the individual profiles varied with the geometric parameters. At a fixed pore-throat diameter, the variation in the average pore diameter with porosity is mild because the average diameter of pore bodies does not vary with the porosity. Increasing the pore-throat diameter increases the average pore diameter, i.e., $d_{p,\text{avg}}=12.5\;\text{nm}$ for the porous medium with $d_{\text{pt}}=5\;\text{nm}$, and $d_{p,\text{avg}}=11.4\;\text{nm}$ for the porous medium with $d_{\text{pt}}=3\;\text{nm}$. The local pore sizes range from 2.5 nm to 27.5 nm (i.e., the local lattice numbers range from 5 to 55 lattices) for all reconstructions, which indicates the existence of multiple transport mechanisms in nanoscale pore spaces.

**Fig. 5:**
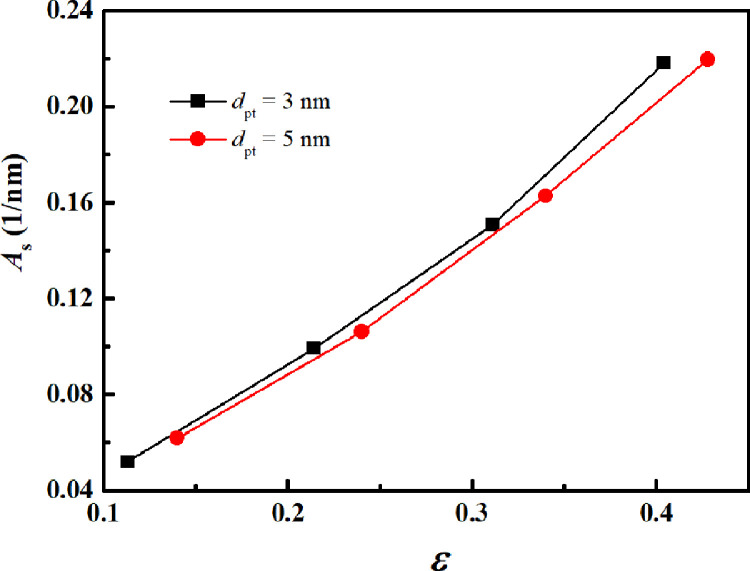
**Variation of the specific surface area with porosity for the pore-throat nanoporous media**.

Because the specific surface area greatly affects the degree of gas-solid interaction (i.e., slip velocity), this parameter is also characterized for the reconstructed pore-throat nanoporous media. According to the definition of the specific surface area (i.e., the surface area per unit of solid volume for a porous material), the voxels occupied by the gas-solid interfaces and solid skeletons are counted, and their ratio acts as the specific surface area. Fig. 5 displays the variations in the specific surface area ($A_{s}$) with porosity for the reconstructed pore-throat nanoporous media. The specific surface area almost increases linearly with the porosity. At a fixed porosity, a slight increment is observed as the pore-throat diameter increases, suggesting that the increase in pore-throat diameter has a slight effect on the specific surface area. For example, at the porosity of 0.404, the specific surface area for $d_{\text{pt}}=3\;\text{nm}$ is higher by 5.5 % than that for $d_{\text{pt}}=5\;\text{nm}$, because the pore size in $d_{\text{pt}}=3\;\text{nm}$ is generally smaller than that in $d_{\text{pt}}=5\;\text{nm}$ (Fig. 4).

### 4.3 Governing equations and boundary conditions

Because LBMs mimic an unsteady process, the governing equations for the viscous flow and mass diffusion under the unsteady state are presented in this section. By separately solving the governing equations through the pore-scale LBMs, the apparent permeability and diffusivity of nanoporous media can be obtained.

#### 4.3.1 Gas viscous flow in nanoporous media

The governing equations (i.e., continuity and N-S equations) for the unsteady viscous flow of gas in the void pore spaces of the nanoporous media are given by:

(21a) ∇·u=0,

(21b) $$\frac{\partial \left(\rho u\right)}{\partial t}+\nabla \cdot \left(\rho \mathrm{uu}\right)=-\nabla p+\mu \nabla^{2}u$$

where **u** is the velocity vector, $u=(u_{x},u_{y},u_{z})$. The internal term (i.e., the second term on the left-hand side) in Eq. 21b is negligible because of the extremely low velocity caused by the nanoscale pore size and small pressure gradient. The pressure gradient and viscous force terms on the right-hand side are dominant under the steady state. Under the initial condition (t=0), u=0. When t>0, the boundary conditions for the gas viscous flow are:

(22) $$\left\{ \begin{array}{c} p=p_{\text{in}},x=0,\;t>0\\ p=p_{\text{out}},x=L_{x},\;t>0\\ u_{x}=u_{s,x},u_{z}=u_{s,z},u_{y}=0,y=0\;\text{or}\;L_{y},\;t>0\\ u_{x}=u_{s,x},u_{y}=u_{s,y},u_{z}=0,z=0\;\text{or}\;L_{z},\;t>0\\ u\left(x_{s}\right)=u_{s},\;x_{s}\in \Gamma \end{array}\right.$$

where $x_{s}$ are the nodes on the pore surfaces; Γ denotes the pore surfaces; and $u_{s}$ is the slip velocity on the pore surface, $u_{s}=\left(u_{s,x},u_{s,y},u_{s,z}\right)$.

#### 4.3.2 Gas mass diffusion in nanoporous media

The governing equation for the unsteady diffusion of gas in the void pore spaces of nanoporous media is given by:

(23) $$\frac{\partial C}{\partial t}-\nabla \cdot \left(D_{\alpha }\nabla C\right)=0$$

where $D_{\alpha }$ denotes the local diffusivity. The calculation of $D_{\alpha }$ has been well documented in a previous work [36]. Under the initial condition (t=0), C=0. When t>0, the boundary conditions for mass diffusion are:

(24) $$\left\{ \begin{array}{c} C=C_{\text{in}},x=0,\;t>0\\ C=C_{\text{out}},x=L_{x},\;t>0\\ \partial C/\partial y=0,y=0\;\text{or}\;L_{y},\;t>0\\ \partial C/\partial z=0,z=0\;\text{or}\;L_{z},\;t>0 \end{array}\right.$$

Note that in Eq. 24, the concentrations at the inlet and outlet were acquired from the pressure boundary conditions used in Eq. 22 to ensure the same working conditions.

### 4.4 Pore-scale lattice Boltzmann methods

To predict the gas transport properties through nanoporous media, a powerful approach is required to solve the aforementioned governing equations at the pore scale, and then obtain the gas transport coefficients (i.e., apparent permeability and apparent diffusivity). In this study, pore-scale LBMs are employed owing to their success in handling irregular boundary conditions at the microscopic level [31,35]. To reproduce the viscous flow process, the flow governing equations (Eq. 21 are solved by the LEV-LBM with the multiple relaxation time collision operator [27]. To reproduce the mass diffusion, the diffusion governing equation (Eq. 23) is solved by the LD-LBM with the single relaxation time collision operator [36]. Because the model of nanoporous media is built in the 3D pore space with irregular surfaces, the D3Q19 discrete velocity scheme in LBMs is used to ensure a high degree of accuracy [6]. LBM mimics the particle propagation and collision at local lattice sites rather than discrete the governing equations directly. Thus, five lattices (i.e., gird mesh) in LBM are enough to resolve the velocity profile in a single nanopore [31,48]. In the present work, the pore sizes range from 2.5 nm to 27.5 nm in the reconstructions (Fig. 4), which corresponds to the lattice numbers ranging from 5 to 55 lattices (because the lattice resolution is 0.5 nm per lattice). Thus, the lattice number in all the computational domains meets the requirement of the grid mesh independence solution.

In the LEV-LBM, the combined bounce-back and diffuse reflection boundary is imposed on the gas-solid interfaces to consider the slip effect, and the local effective relaxation time is introduced to consider the spatially varying viscosity in the void spaces of nanoporous media. This treatment has proved reliable in capturing the inhomogeneous in the nanoscale pores [27]. The pressure boundaries at the inlet and outlet are imposed via the method proposed by Hecht and Harting [49]. In the LD-LBM, the bounce-back boundary condition is imposed on the gas-solid interfaces to achieve impermeable boundaries. The local diffusivity is introduced to consider the multiple diffusion mechanisms. The concentration boundaries at the inlet and outlet are imposed via the non-equilibrium bounce-back proposed by Zou and He [50]. Detailed descriptions of the LBM approaches can be found in our previous work [6,27,36].

The macroscopic variables [e.g., velocity vector, $u=(u_{x},u_{y},u_{z})$ and mass flux vector, $J=(J_{x},J_{y},J_{z})$] can be acquired once the LBM simulations meet the convergence criteria for the steady state. With the above variables, the desired transport coefficients, namely, the apparent permeability ($k_{\mathrm{app}}$) and apparent diffusivity ($D_{\text{app}}$), are obtained by applying Darcy's law (Eq. 25) and Fick's first law (Eq. 26) as follows:

(25) $$k_{\mathrm{app}}=-\frac{\mu_{0}\langle u_{x}\rangle }{dp/dx},$$

(26) $$D_{\mathrm{app}}=-\frac{\langle J_{x}\rangle }{\mathrm{dC}/\mathrm{dx}}$$

where 〈〉 denotes the volume average based on the entire computation domain; $u_{x}$ and $J_{x}$ denote the velocity and mass flux along the flow and diffusion directions (*x*-direction), respectively. The pressure (*p*) is a foregone parameter determined by the working conditions, as mentioned previously. The real gas effect is considered through the *Kn*, in which the real gas EOS is adopted to calculate the number density (Eq. 9), as indicated in [27,51]. Furthermore, the bulk dynamic viscosity ($\mu_{0}$) of methane gas is calculated by the classic correlation developed by Lee et al. [52], in which $\mu_{0}$ varies with average pressure. Substituting these variables into Eq. 6, the diffusion-flow ratio for the nanoporous media can be obtained.

## 5 Results and discussion

### 5.1 Methodology verification

#### 5.1.1 Gas viscous flow with the slip effect

The LEV-LBM, which is used to predict the apparent permeability of the nanoporous media, is validated by classical analytical solutions of the gas viscous flow in nanotubes in our previous work [27]. The validation results demonstrate that the LEV-LBM works well for a wide range of *Kn* flow regimes. To avoid redundancy, the validation process is not repeated here. The detailed model validation can be found in our previous work [27].

#### 5.1.2 Gas diffusion with the multiple diffusions

**Fig. 6:**
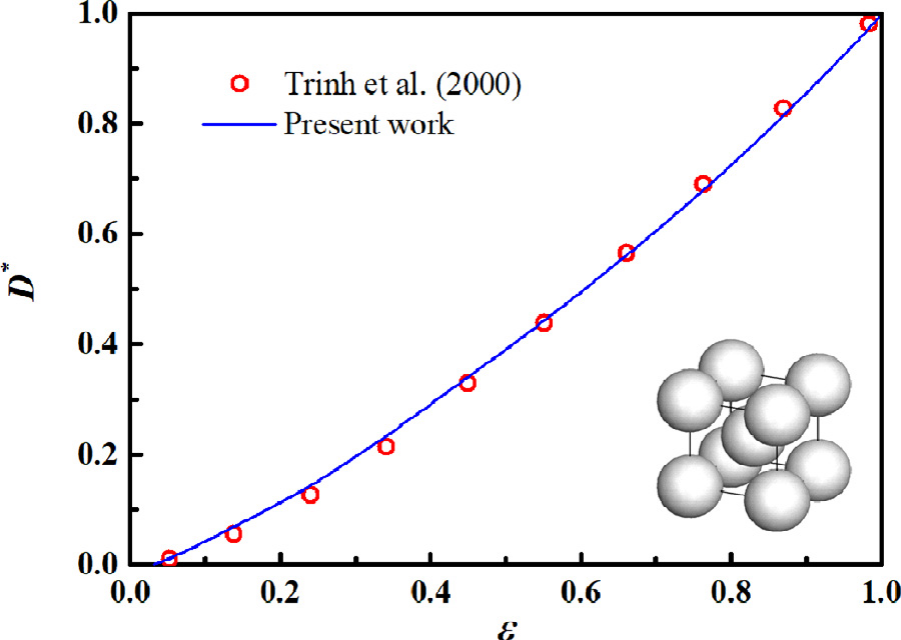
**Normalized diffusivity varying with porosity for a body-centered cubic unit**.

The LD-LBM, which is used to predict the apparent diffusivity of the nanoporous media, is an extension of our previous model [36]. In our previous work [36], the LB algorithm is validated for 2D porous media. To validate its feasibility for 3D porous media, the gas flow in a body-centered cubic unit spanning a wide range of porosities is simulated. According to the previous work [36], the gas diffusion in the pore spaces is assumed to be the pure molecular diffusion; the calculated diffusivity of porous media, which is normalized by the molecular diffusivity in the pore spaces, is the normalized diffusivity ($D^{*}$). The normalized diffusivity is a dimensionless parameter ($0\leq D^{*}\leq 1$), relying only on the pore structures. Fig. 6 shows the variations in the normalized diffusivity with porosity. As the porosity increased, the normalized diffusivity calculated by the LD-LBM agreed well with the Monte Carlo simulation results reported by Trinh et al. [53]. This finding indicates the feasibility of the present LD-LBM for 3D porous media.

### 5.2 Gas transport in the simple nanopores

**Fig. 7:**
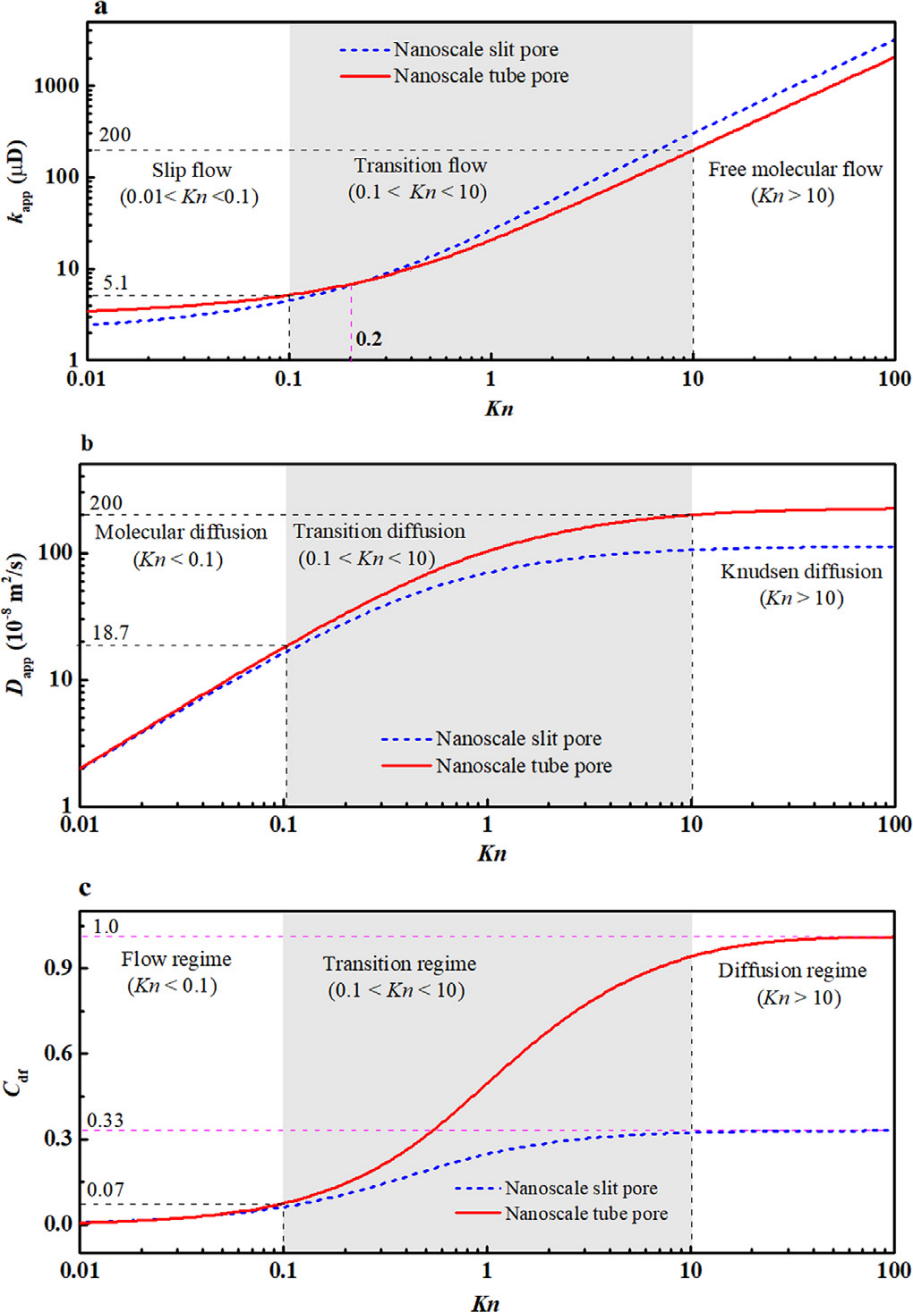
**Variation of transport coefficients with the *Kn* in simple nanoscale pores**. (a) Apparent permeability; (b) Apparent diffusivity; (c) Diffusion-flow ratio.

Slit and tube pores are classical models that have been widely used to explore gas transport behaviors at the nanoscale, and are also analyzed here. Methane is adopted as the working fluid. Given that the present work focuses on the nanoscale transport behaviors, the temperature and characteristic size are fixed at 350 K and 5 nm, according to Ref. [36]. Varying Kn can be achieved by adjusting the average pressure. The apparent permeability ($k_{\text{app}}$), apparent diffusivity ($D_{\text{app}}$), and diffusion-flow ratio ($C_{\text{df}}$) in the nanoscale slit or tube can then be obtained through the following formulas: Eqs. 7 and 13 for $k_{\text{app}}$; Eqs. 14 and 15 for $D_{\text{app}}$; and Eqs. 19 and 20 for $C_{\text{df}}$. Fig. 7a–c show the variations of these transport coefficients ($k_{\text{app}}$, $D_{\text{app}}$, and $C_{\text{df}}$) with Kn (0.01<Kn<100) in the logarithmic coordinate.

In Fig. 7a, $k_{\text{app}}$ exponentially increases with Kn in the nanoscale slit and tube pores. As *Kn* increases from 0.01 to 100, the gas flow behaviors fall into three flow regimes: slip flow (0.01<Kn<0.1), transition flow (0.1<Kn<10), and free molecular flow (Kn>10), according to the magnitude of *Kn* [54]. In the slip flow regime (0.01<Kn<0.1), $k_{\text{app}}$ increases slowly because the contribution from the second-order *Kn* term to $k_{\text{app}}$ is mild at a small *Kn* Eqs. 7 and 13. However, when Kn>0.1, $k_{\text{app}}$ rapidly increases, and the second-order *Kn* term becomes significant. Specifically, in the transition regime (0.1<Kn<10), $k_{\text{app}}$ increases from 5.1 μD to 200 μD for the nanoscale tube pore, an increment of approximately two orders of magnitude (∼ 39 times). This indicates a significant slip effect at the large *Kn*. Furthermore, it can be observed that *Kn* = 0.2 is the turning point for the nanoscale slit and tube pores. When *Kn* < 0.2, the $k_{\text{app}}$in the slit pore is smaller than that in the tube pore. When *Kn* > 0.2, the $k_{\text{app}}$ shows the opposite result, which indicates that the pore shape plays a key role in determining $k_{\text{app}}$. When Kn<0.01, a lower bound (the minimum $k_{\text{app}}$) exists for both slit and tube pores, where the slip effect almost disappears. This finding is consistent with the previous work [55], where the correction factor for the slip effect is close to one (means no slip effect) at *Kn* < 0.01 in a nanoscale slit pore. In fact, the value of the lower bound is equal to the absolute permeability ($k_{D}$). According to Eqs. 7 and 13, it can be deduced that $k_{D}$ is equal to $H^{2}/12$ and $R^{2}/8$ for the slit and tube pores, respectively.

In Fig. 7b, $D_{\text{app}}$ increases with Kn in a logarithmic trend, which differs from the trend in $k_{\text{app}}$ (the exponential trend in Fig. 7a). Nevertheless, variations in $D_{\mathrm{app}}$ can be also divided into three regimes according to *Kn*: molecular diffusion (Kn<0.1), transition diffusion (0.1<Kn<10), and Knudsen diffusion (Kn>10). In the molecular diffusion regime (Kn<0.1), $D_{\mathrm{app}}$ increases rapidly, and the differences between the slit and tube pores are slight. This is because molecular diffusion is dominant (i.e., $D_{\mathrm{app}}\to D_{M}$) at a small *Kn*, and $D_{M}$ is independent of the pore shape (see Eq. 16)*.* In the transition diffusion regime (0.1<Kn<10), $D_{\mathrm{app}}$ increases from 18.7 × 10^−8^ m^2^/s to 200 × 10^−8^ m^2^/s (in the tube pore, for example), an increment of approximately one order of magnitude (∼11 times). Furthermore, in this regime, $D_{\mathrm{app}}$ in the tube pore is larger than that in the slit pore. Their differences increase with *Kn* because $D_{\mathrm{app}}$ is inversely proportional to *Kn* by the distinct scale factors that varied with the pore shape Eqs. 14 and 15. The increment in $D_{\text{app}}$ (∼11 times) is significantly lower than that in $k_{\text{app}}$ (∼39 times) because the slip effect is more sensitive to *Kn*. In the Knudsen diffusion (Kn>10), $D_{\text{app}}$ increases slowly; and $D_{\text{app}}$in the tube pore is higher than that in the slit pore. This occurs because the Knudsen diffusion is dominant (i.e., $D_{\mathrm{app}}\to D_{\mathrm{Kn}}$) at a large *Kn*, and the $D_{\mathrm{Kn}}$ in the tube pore ($2R\bar{v}/3$) is larger than that in the slit pore ($H\bar{v}/3$). In addition, an upper bound (i.e., maximum $D_{\text{app}}$) exists for both slip and tube pores, whose value is equal to the Knudsen diffusivity ($D_{\mathrm{Kn}}$).

In Fig. 7c, $C_{\text{df}}$ increases with Kn. The variation of $C_{\text{df}}$ is divided into three regimes according to *Kn*: flow regime (Kn<0.1), transition regime (0.1<Kn<10), and diffusion regime (Kn>10). In the flow regime (Kn<0.1), $C_{\text{df}}$ increases slowly with *Kn*. Its value is less than 0.07, indicating that viscous flow is the dominant transport mechanism. In this situation, $C_{\text{df}}$ in the tube and silt pores are almost the samebecause the key parameters in determining $C_{\text{df}}$ are similar. In the transition regime (0.1<Kn<10), $C_{\text{df}}$ increases rapidly, and the increasing rates for the tube and slit pores are distinct (i.e., the tube pore being faster than the slit pore). This occurs because the parameters ($k_{\text{app}}$ and $D_{\text{app}}$) in determining $C_{\text{df}}$ are sensitive to the pore shape (Fig. 7a,b). In the diffusion regime (Kn>10), $C_{\text{df}}$ increases slowly and approaches a plateau (i.e., the maximum $C_{\text{df}}$), where the maximum $C_{\text{df}}$ is 1.0 and 0.33 for the tube and slit pores, respectively. Meanwhile, the differences between the slit and tube pores are significant because in this regime, the gas-wall collisions are significant and the gas transport is likely to be Knudsen diffusion (whose diffusivity related to the pore shape). The above findings suggest that the dominant transport mechanism in single nanoscale pores is viscous flow, and its contribution to gas transport capacity is dependent on the chosen geometric model and *Kn*.

### 5.3 Apparent permeability of the nanoporous media

The apparent permeability ($k_{\text{app}}$) is a crucial parameter to gauge the gas transport ability through a nanoporous medium via the viscous flow and the slip effect. In this section, $k_{\text{app}}$ in the pore-throat nanoporous media (Fig. 3) is calculated using the LEV-LBM. Methane is adopted as the working fluid. The temperature is fixed at 350 K, which is identical to the setting in simple nanopore models. However, because*Kn* in nanoporous media varies spatially, an average Knudsen number ($Kn_{\text{avg}}$) is adopted [56], which is defined as the ratio of the mean free path ($\lambda_{0}$) and the average pore diameter of nanoporous structures ($d_{p,\mathrm{avg}}$), expressed as:

(27) $$Kn_{\text{avg}}=\frac{\lambda_{0}}{d_{p,\mathrm{avg}}}$$

**Fig. 8:**
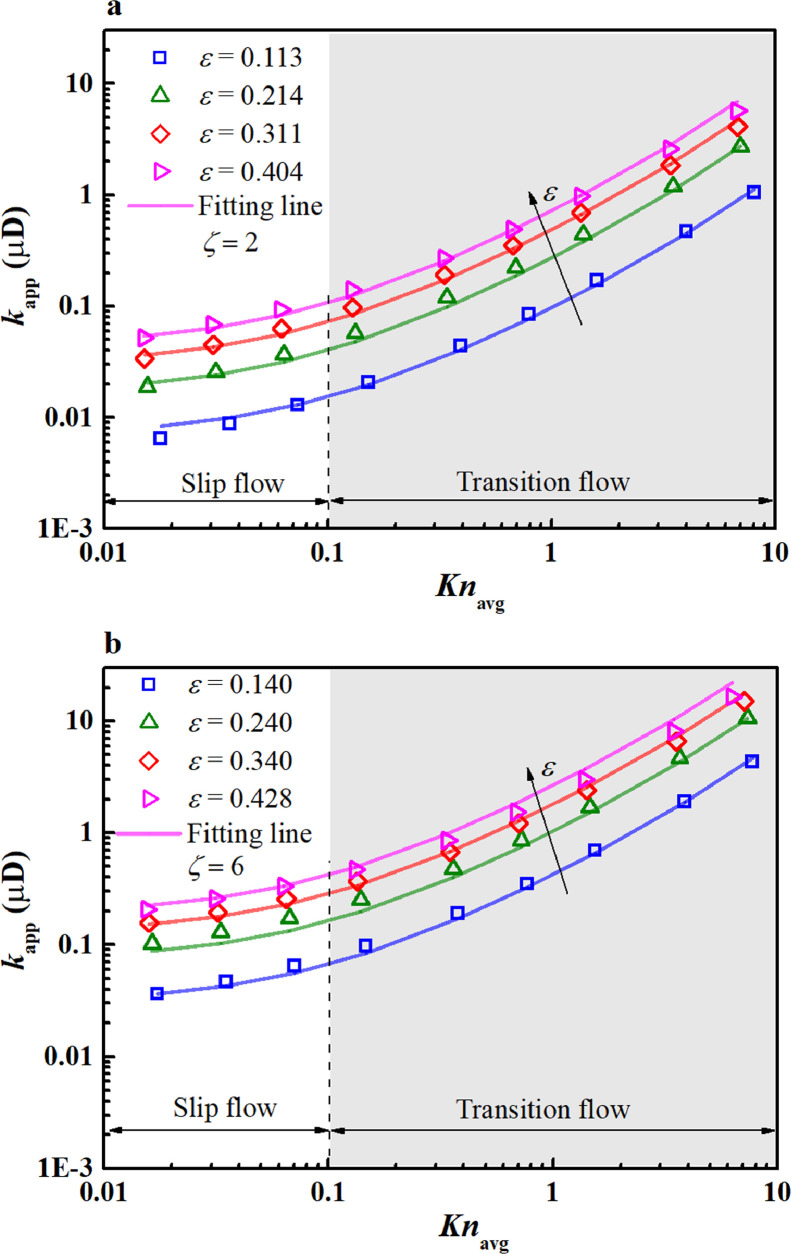
**Variation of the apparent permeability with *Kn*_avg_**. (a) $d_{\text{pt}}=3\text{nm}$; (b) $d_{\text{pt}}=5\text{nm}$.

In Eq. 27, $d_{p,\mathrm{avg}}$ is fixed for a given nanoporous structure (Fig. 4); and $\lambda_{0}$ is associated with the gas type, temperature, and pressure (Eq. 9). The varying $Kn_{\text{avg}}$ is thus achieved by adjusting the average pressure (0.1–50 MPa) with respect to the methane gas at 350 K. Fig. 8 shows the variations of $k_{\text{app}}$ with $Kn_{\text{avg}}$ and porosity in the dual logarithmic coordinate.

In Fig. 8a, $k_{\text{app}}$ increases exponentially with $Kn_{\text{avg}}$, which is similar to the trend in simple nanopores (Fig. 7a). The variations of $k_{\text{app}}$ fall into two flow regimes (i.e., slip and transition flow regimes), when $Kn_{\text{avg}}$ ranges from ∼0.01 to ∼10. In the slip flow regime ($0.01<Kn_{\mathrm{avg}}<0.1$), $k_{\text{app}}$ increases relatively slowly; in the transition flow regime ($0.1<Kn_{\mathrm{avg}}<10$), $k_{\text{app}}$ increases rapidly. At ε=0.113, $k_{\text{app}}$ increases from 0.016 μD to 1.054 μD (∼66 times). $Kn_{\text{avg}}$ varies from 0.1 to 8.1, implying that the increment of $k_{\text{app}}$ is approximately two orders of magnitude in the transition flow regime. This kind of variation is in line with that of the single nanopore; hence, the interoperations are similar. However, the absolute values of $k_{\text{app}}$ in the nanoporous media are smaller (<20 μD). This is attributed to the small pore-throat diameter (*d*_pt_ = 3 or 5 nm) as well as the tortuous pathways in the nanoporous media that cause a large transport resistance. Furthermore, the increase in porosity enhanced $k_{\text{app}}$. At *Kn*_avg_ = 0.1, the increase in porosity from 0.113 to 0.404 enhances $k_{\text{app}}$ from 0.016 µD to 0.112 µD (∼7 times). A high porosity means abundant void spaces that allow the gas molecules to pass through easily.

In Fig. 8b, the variations of $k_{\text{app}}$ with $Kn_{\text{avg}}$ and ε are similar to those discussed in Fig. 8a. However, $k_{\text{app}}$ in Fig. 8b, with $d_{\text{pt}}=5\text{nm}$ is significantly larger than that in Fig. 8a with $d_{\text{pt}}=3\text{nm}$, indicating that increasing the pore-throat diameter improves $k_{\text{app}}$. This occurs because the pore-throat is a bottleneck, and its increase can greatly facilitate gas transport. These findings indicate the significance of the geometric structure on the gas transport ability.

To estimate $k_{\text{app}}$ in the pore-throat nanoporous media, a fitting correlation (Eq. 28) is proposed based on the present LEV-LBM simulation results (72 data points in Fig. 8). In Eq. 28, $k_{\text{app}}$ is proportional to the second-order *Kn*_avg_, which also explains the rapid rise of $k_{\text{app}}$ within the transition flow regime, as shown in Fig. 8:

(28) $$k_{\text{app}}=\zeta \frac{\varepsilon d_{p,\text{avg}}^{2.4}}{{\left[1-0.28\ln\left(\varepsilon \right)\right]}^{2}}\left(1+12Kn_{\text{avg}}+Kn_{\mathrm{avg}}^{2}\right).$$

The fitting results from Eq. 28 are plotted in Fig. 8a,b, both of which show good agreement with the simulation results over a wide range of $Kn_{\text{avg}}$. The mean relative errors from Eq. 28 are 9.1% and 10.9% for $d_{\text{pt}}=3\text{nm}$and $d_{\text{pt}}=5\text{nm}$, respectively, which are small enough to meet the practical application requirements.

When applying Eq. 28 to predict $k_{\text{app}}$, the only required parameters are the porosity (ε), average pore diameter ($d_{p,\text{avg}}$), average Knudsen number ($Kn_{\text{avg}}$), and correction coefficient for the pore-throat diameter (ζ). Among them, ε and $d_{p,\text{avg}}$ are dependent on the geometric structure; $Kn_{\text{avg}}$ is related to the pressure, temperature, and gas species; and ζ is related to the pore-throat diameter. In the present study, ζ is fitted from the simulation results, i.e., ζ=2 for $d_{\text{pt}}=3\text{nm}$; ζ=6 for $d_{\text{pt}}=5\text{nm}$. With the above parameters, $k_{\text{app}}$ in pore-throat nanoporous media can be easily estimated using Eq. 28 at $Kn_{\text{avg}}<10$. It should be noted that the results at *Kn*_avg_ >10 are not displayed because of the limited application scope of the LEV-LBM. Further study is required to obtain $k_{\text{app}}$ at *Kn*_avg_ >10.

**Fig. 9:**
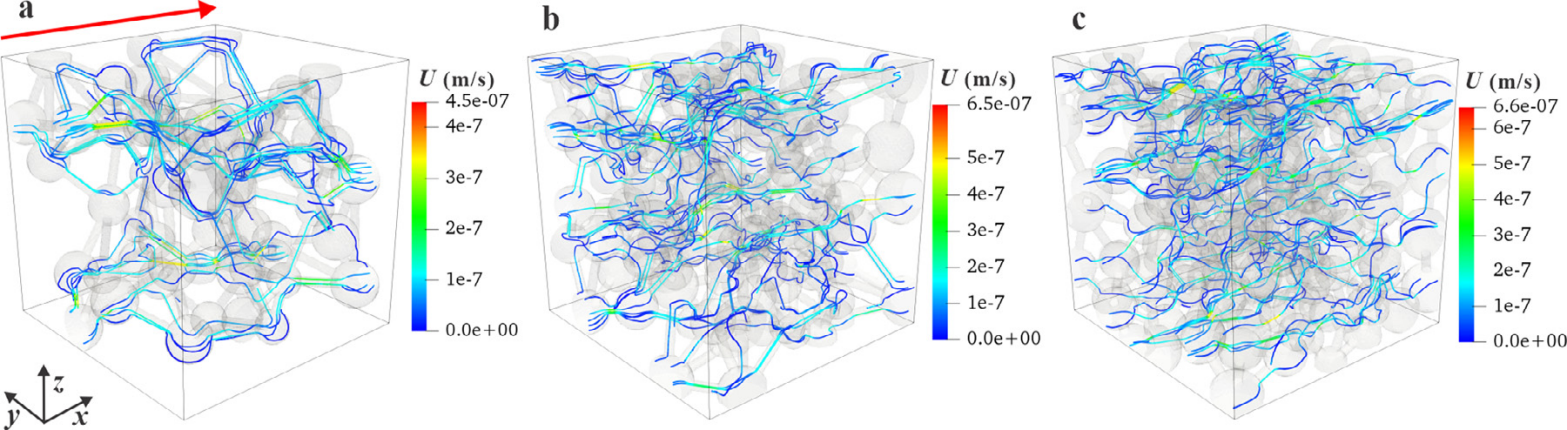
**Velocity distribution and streamlines**. (a–c) Three nanoporous media correspond to the reconstructions in Fig. 3(a–c) at *p* = 1 MPa. Red and blue in the legend represents high and low velocity, respectively.

Velocity distribution and streamlines are common methods to visualize gas flow behaviors. The dense streamlines means the high velocity. To visualize the spatial velocity distribution, Fig. 9 depicts the velocity distributions and streamlines in three nanoporous media that correspond to the reconstructions in Fig. 3a–c at *p* = 1 MPa. The streamlines in the high-porosity nanoporous medium (ε=0.311 in Fig. 9c) are less tortuous than those in the low-porosity nanoporous medium (ε=0.113 in Fig. 9a). The increase in porosity enhances the gas transport spaces, providing the gas molecules with more viable flow pathways, which also increases the $k_{\text{app}}$ with porosity, as shown in Fig. 8. Moreover, the velocity (represented by the colorful streamlines) in pore throats is higher than that in the pore bodies because the total mass flow rate is conserved along the flow direction. The absence of streamlines in some pore spaces is due to the extreamly low velocity locally.

### 5.4 Apparent diffusivity of the nanoporous media

**Fig. 10:**
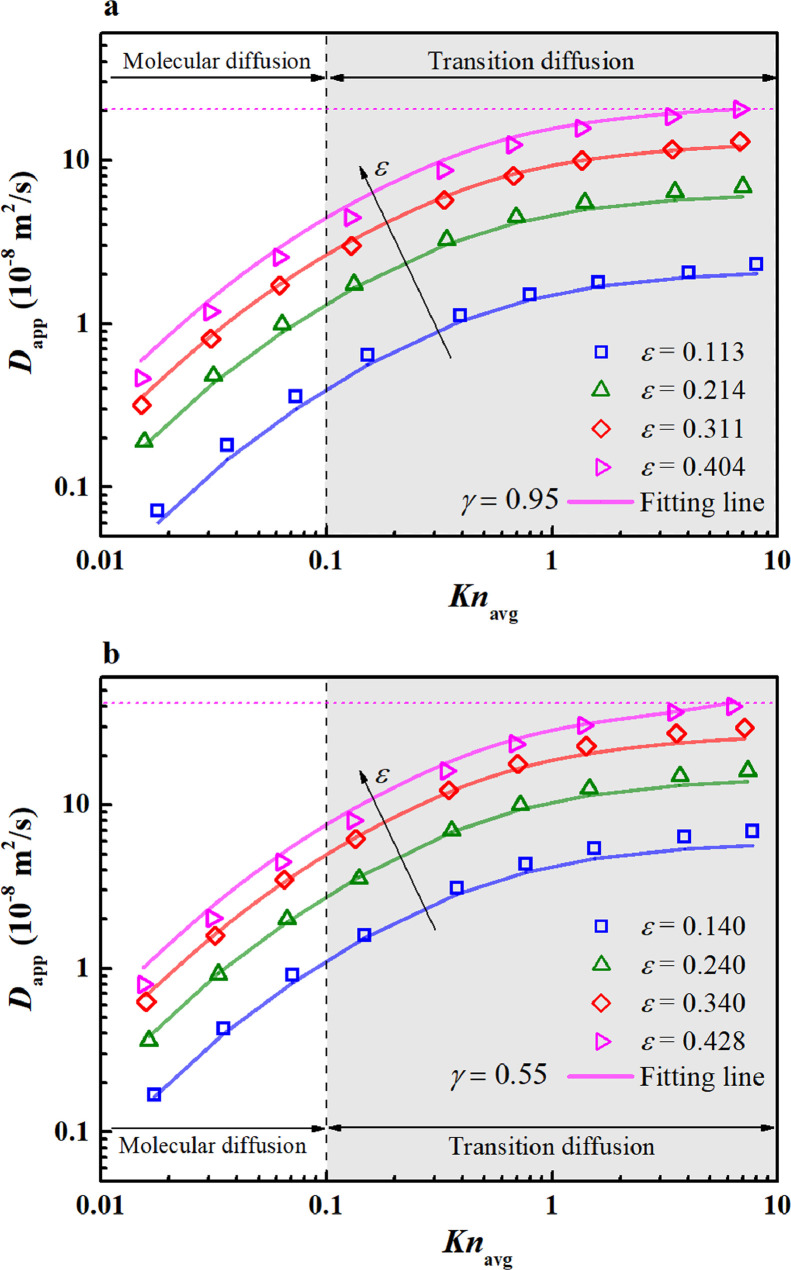
**Variation of the apparent diffusivity with the *Kn*_avg_**. (a) $d_{\text{pt}}=3\text{nm}$; (b) $d_{\text{pt}}=5\text{nm}$.

The apparent diffusivity ($D_{\text{app}}$) is a crucial parameter to quantify the gas transport ability through nanoporous media via mass diffusion along with the multiple diffusion effect. In this section, $D_{\text{app}}$ is calculated for the reconstructed pore-throat nanoporous media by the LD-LBM, with the working fluid, temperature, and pressure identical to those of the gas viscous flow in Section 5.2. Fig. 10 shows that the calculated $D_{\text{app}}$varies with $Kn_{\text{avg}}$ in the dual logarithmic coordinate.

In Fig. 10a, when $Kn_{\text{avg}}$ varies from ∼ 0.01 to ∼ 10, $D_{\text{app}}$ increases with $Kn_{\text{avg}}$ in a logarithmic trend in the dual logarithmic coordinate. This increasing trend is similar to that of simple nanopores (Fig. 7b). Likewise, variations of $D_{\text{app}}$ are described separately within the molecular and transition diffusion regimes. In the molecular diffusion regime ($Kn_{avg}<0.1$), $D_{\text{app}}$ rapidly increases; in the transition diffusion regime ($0.1<Kn_{avg}<10$), $D_{\text{app}}$ increases slowly. At ε=0.113, $D_{\text{app}}$ increases from 0.39 × 10^−8^ m^2^/s to 2.30 × 10^−8^ m^2^/s (∼6 times) when $Kn_{\text{avg}}$ increases from 0.1 to 8.1. This increment is approximately one order of magnitude. The increment in $D_{\text{app}}$ is weaker (one order of magnitude) than that in $k_{\text{app}}$ (two orders of magnitudes) under the same conditions ($0.1<Kn_{avg}<8.1$, ε=0.113). The reason is because $k_{\text{app}}$ is proportional to the second-order of $Kn_{\text{avg}}$ (Eq. 28), whereas $D_{\text{app}}$ is inversely proportional to the first-order of $Kn_{\text{avg}}$ (Eq. 29). When is $Kn_{\text{avg}}$ close to 10, $D_{\text{app}}$ approaches a plateau (i.e., the maximum value). In this situation, the maximum $D_{\text{app}}$ is numerically equal to the Knudsen diffusivity ($D_{\text{app}}\to D_{\text{Kn}}$). The nanoporous medium with the highest porosity and largest pore size will have the largest $D_{\text{app}}$ because the Knudsen diffusivity is proportional to the pore size (e.g., ε=0.404). Because the variation of $D_{\text{app}}$ in nanoporous media is consistent with the single nanopore results shown in Fig. 7b, they share similar interoperations. Nevertheless, the absolute values of $D_{\text{app}}$ in the nanoporous media are smaller (<40 × 10^−8^ m^2^/s) than those in single nanopores because of the high transport resistance caused by the solid skeletons. Increasing porosity also improves $D_{\text{app}}$ because the ample void spaces due to the high porosity result in gas diffusion. For example, when the porosity increases from 0.113 to 0.404 at *Kn*_avg_ = 0.1, $D_{\text{app}}$ increases from 0.39 × 10^−8^ m^2^/s to 4.51 × 10^−8^ m^2^/s, an increment of approximately 11.6 times.

In Fig. 10b, the variations of $D_{\text{app}}$ with $Kn_{\text{avg}}$ and ε are similar to those discussed in Fig. 10a. However, $D_{\text{app}}$ in Fig. 10b with $d_{\text{pt}}=5\text{nm}$ is slightly higher than that in Fig. 10a with $d_{\text{pt}}=3\text{nm}$, indicating that increasing the pore-throat diameter improves $D_{\text{app}}$. The reasons are the same as those discussed in Fig. 8b.

To estimate $D_{\text{app}}$ in the pore-throat nanoporous media, a fitting correlation Eq. 29) is proposed based on the LD-LBM simulation results (72 data points in Fig. 10). This correlation is inversely proportional to $Kn_{\text{avg}}$, which is analogous to the single pore formula Eqs. 14 and 15.

(29) $$D_{\text{app}}=\frac{\varepsilon }{{\left[1-\gamma \ln\left(\varepsilon \right)\right]}^{2}}\frac{D_{M}}{1+2.5Kn_{\text{avg}}}$$

The fitting results from Eq. 29 are plotted in Fig. 10a,b, which show good agreement with the simulation results over a wide range of $Kn_{\text{avg}}$. The mean relative errors from Eq. 29 are 10.1% and 8.9% for $d_{\text{pt}}=3\;\text{nm}$ and $d_{\text{pt}}=5\;\text{nm}$, respectively, which are suitable for practical applications. When applying Eq. 29 to predict $D_{\text{app}}$, the only required parameters are the porosity (ε), molecular diffusivity ($D_{M}$), average Knudsen number ($Kn_{\text{avg}}$), and correction coefficient for the pore-throat diameter (γ). Among these, ε is dependent on the geometric structure; $D_{M}$ and $Kn_{\text{avg}}$ are related to the working conditions (pressure, temperature, and gas species); and γ is related to the pore-throat diameter. In the present study, γ is fitted from the simulation results, i.e., γ=0.95 for $d_{\text{pt}}=3\;\text{nm}$; and γ=0.55 for $d_{\text{pt}}=5\;\text{nm}$. With the above parameters, $D_{\text{app}}$ in pore-throat nanoporous media can be easily estimated by Eq. 29.

The concentration field can reflect the influence of porous structure features on the diffusion property. To reveal this influence, the concentration is normalized by the contribution difference, which is a dimensionless concentration $C^{*}$ given by:

(30) $$C^{*}=\frac{C-C_{\mathrm{out}}}{C_{\mathrm{in}}-C_{\mathrm{out}}}.$$

**Fig. 11:**
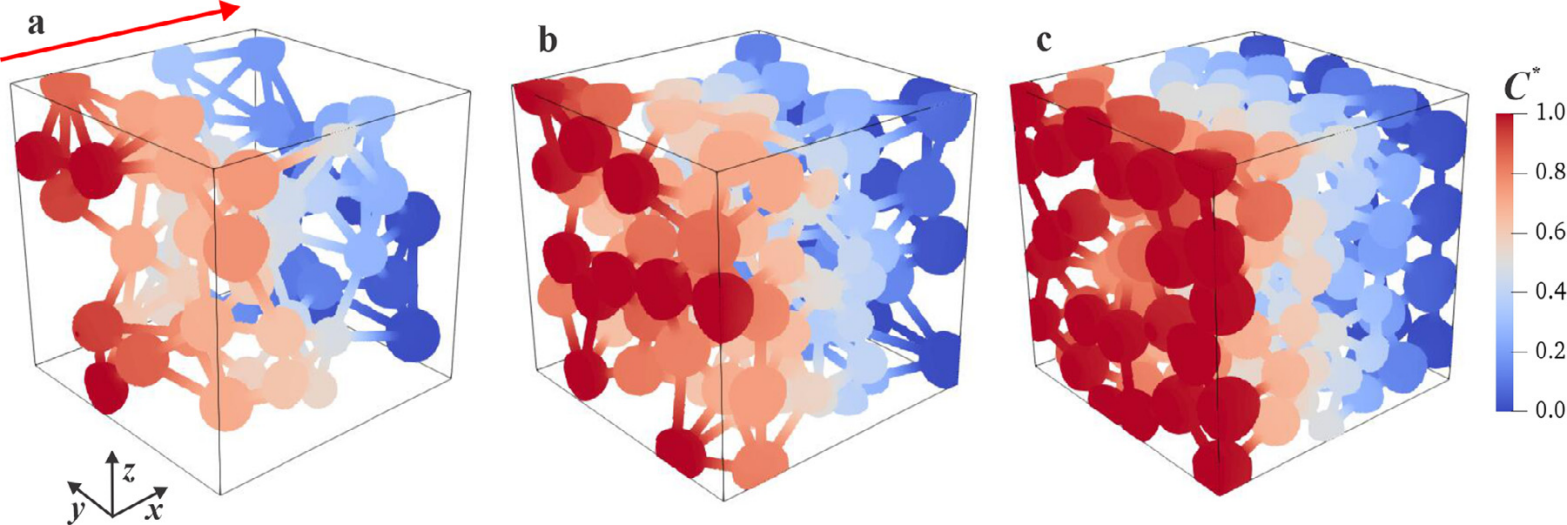
**Distributions of dimensionless concentrations**. (a-c) Three nanoporous media correspond to the reconstructions in Fig. 3(a–c) at *p* = 1 MPa.

**Fig. 12:**
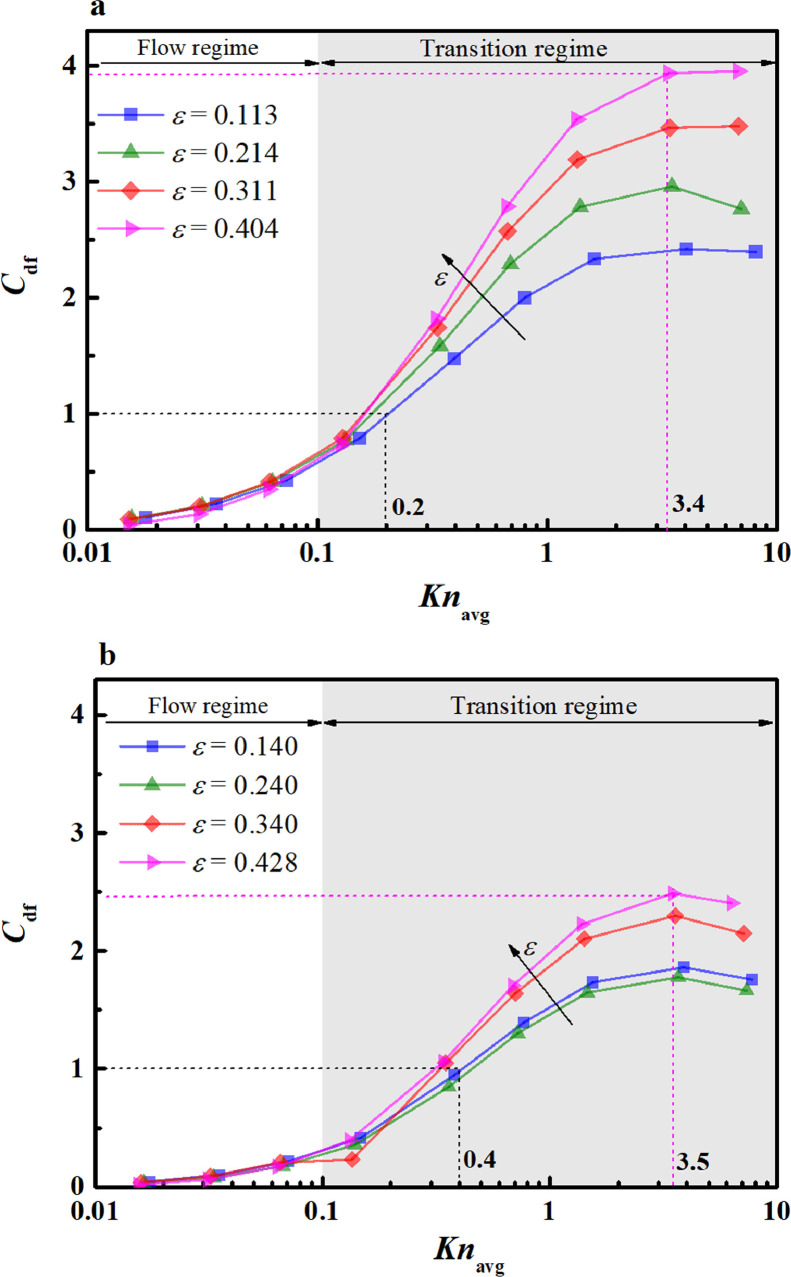
**Variation of diffusion-flow ratio with the *Kn*_avg_**. (a) $d_{\text{pt}}=3\text{nm}$; (b) $d_{\text{pt}}=5\text{nm}$.

The distributions of $C^{*}$ in three nanoporous media are visualized in Fig. 12, which correspond to the reconstructions in Fig. 3a–c at *p* = 1 MPa. It is apparent that $C^{*}$ decreased linearly along the diffusion direction (*x*-direction), obeying the physical characteristics of mass diffusion. As the porosity increases from the left (ε=0.113 in Fig. 11a) to right (ε=0.311 in Fig. 11b), the concentration contours (i.e., the iso-surfaces perpendicular to the diffusion direction) appears more uniform because of the reduced transport resistance at high porosity, which is consistent with the discussion on$D_{\text{app}}$ in Fig. 10.

### 5.5 Diffusion-flow ratio in the nanoporous media

To identify the dominant transport mechanism from the mass diffusion and viscous flow in the reconstructed nanoporous media, the diffusion-flow ratio ($C_{\text{df}}$) is calculated. The working fluid and conditions are the same as those in Sections 5.2 and 5.3. Fig. 12 displays the variation in $C_{\text{df}}$ with $Kn_{\text{avg}}$ in the semi-logarithmic coordinate.

In Fig. 12a, the variations in $C_{\text{df}}$ include the flow and transition regimes. In the flow regime ($0.01<Kn_{avg}<0.1$), $C_{\text{df}}$ increases slowly with increasing $Kn_{\text{avg}}$, which is in line with the simple nanopore results in Fig. 7c. In the transition regime ($0.1<Kn_{\text{avg}}<10$), $C_{\text{df}}$ exhibits an increasing trend. When $0.1<Kn_{\text{avg}}<3.4$, $C_{\text{df}}$ rapidly increases and exceeds the critical point ($C_{\text{df}}=1$) at *Kn*_avg_ = 0.2, above which the mass diffusion is dominant. When$3.4<Kn_{\text{avg}}<10$, $C_{\text{df}}$ gradually increases and reaches the maximum value (∼4) at *Kn*_avg_ = 3.4 for ε=0.404. This variation differs from the simple nanopore results, in which the maximum $C_{\text{df}}$ is less than one within the entire *Kn* regime. This is because $k_{\text{app}}$ is more sensitive to the solid skeleton of porous media than $D_{\text{app}}$; hence, the reduction caused by solid skeletons in $k_{\text{app}}$ is more severe than that in $D_{\text{app}}$. Because $C_{\text{df}}$ is proportional to $D_{\text{app}}/k_{\text{app}}$ (Eq. 6), $C_{\text{df}}$ in nanoporous media is larger than that in the single nanoscale pore. These findings suggest that the occurrence of a solid skeleton in nanoporous media has a significant impact on the transport behavior, altering the dominant transport mechanism from viscous flow to mass diffusion. In addition, the porosity has a slight influence on $C_{\text{df}}$ at *Kn*_avg_ < 0.2; when *Kn*_avg_ > 0.2, its influence gradually increased. At *Kn*_avg_ = 3.4, a significant increase in $C_{\text{df}}$ with the porosity is observed. The $C_{\text{df}}$ at a high porosity (ε=0.404, $C_{\text{df}}=3.94$) is approximately 1.6 times larger than that at a low porosity (ε=0.113, $C_{\text{df}}=2.42$). This increment is attributed to the transformation of the dominant transport at *Kn*_avg_ > 0.2 to Knudsen diffusion, which is strongly dependent on the porosity. This result indicates the dominant transport mechanism is more likely to change in pore-throat nanoporous media with high porosity. Moreover, $C_{\text{df}}$ starts to decrease at *Kn*_avg_ = 3.4 for ε=0.214 and ε=0.113. This is because the small pores in the two porous structures occupy a large proportion (see the peak at 4 nm in Fig. 4), which results in a high local *Kn* and $k_{\text{app}}$, while $D_{\text{app}}$ approaches a constant ($D_{\text{app}}\to D_{\text{Kn}}$).

In Fig. 12b, the variations of $C_{\text{df}}$ with $Kn_{\text{avg}}$ and ε are almost identical to those in Fig. 12a. Their difference is that the increased pore throat in Fig. 12b) decreases $C_{\text{df}}$. The mass diffusion is dominant when $Kn_{\text{avg}}>0.4$. The maximum $C_{\text{df}}$ was ∼2.4 at $Kn_{\text{avg}}=3.5$ for ε=0.428. When *Kn*_avg_ is greater than 3.5, $C_{\text{df}}$ shows a decreasing trend because of the increase rates in $k_{\text{app}}$ and $D_{\text{app}}$. Actually, $k_{\text{app}}$ increases fast (Fig. 8b), while $D_{\text{app}}$ increases slowly (Fig. 10b) at $Kn_{\text{avg}}>3.5$. This means that the increase rate of $k_{\text{app}}$ is much higher than that of $D_{\text{app}}$. According to the definition of $C_{\text{df}}$ (Eq. 6), $C_{\text{df}}$ is proportional to $D_{\text{app}}$, but inversely proportional to $k_{\text{app}}$. Therefore, the decrease in $C_{\text{df}}$ can be seen in Fig. 12b when *Kn*_avg_ is greater than 3.5. These findings indicate that the magnitude of increase of $C_{\text{df}}$ is significantly related to *Kn*_avg_ and the structural parameters. Moreover, absorbed gas appearing on the pore surfaces will influence the viscous flow and mass diffusion via the transport coefficients (i.e., apparent permeability and apparent diffusivity) and the local nonuniform density distribution for the methane transport in nanoporous media [57,58], which might lead to the alternative of the dominant mechanism. However, the influence of the adsorbed gas is significant in a small pore size (<2 nm) [37], which is beyond the present pore size range (2.5–27.5 nm). Therefore, the adsorbed gas is negligible in the present work.

## 6 Conclusion

In this work, the gas viscous flow and mass diffusion processes in single nanopores are analyzed by a theoretical method, and 3D nanoporous media are simulated through the pore-scale LBM. To determine the dominant transport mechanism, a dimensionless parameter, i.e., the diffusion-flow ratio, is established, which is expressed as a function of the apparent permeability, apparent diffusivity, bulk dynamic viscosity, and working pressure. The results show that the apparent permeability and apparent diffusivity increase with *Kn*_avg_ for nanoporous media(or *Kn* for simple nanopores). When *Kn*_avg_ (or *Kn*) increases from 0.1 to 10, the increment in the apparent permeability is approximately two orders of magnitude, which is more significant than that of the apparent diffusivity (∼one order of magnitude). When *Kn* < 0.01, the apparent permeability has a lower bound whose value is the absolute permeability. When *Kn* > 10, the apparent diffusivity has an upper bound whose value is close to the Knudsen diffusivity. The dominant transport mechanism in single nanoscale pores is the viscous flow, where the maximum value of the diffusion-flow ratio is less than one. In nanoporous media, it relies heavily on *Kn*_avg_ and the structural parameters. For nanoporous media with $d_{\text{pt}}=3\text{nm}$, *Kn*_avg_ = 0.2 is the critical point above which the mass diffusion is dominant; otherwise, the viscous flow is dominant. As *Kn*_avg_ increased to 3.4, the mass diffusion is overwhelming, with the maximum diffusion-flow ratio reaching ∼4. This finding indicates that the solid skeleton of porous media plays a key role in determining the dominant transport mechanism. Considering that the present work is limited to the pore-throat nanoporous media, further work is expected to focus on other more complicated porous media to disclose the influences of morphology and topology on the dominant transport mechanism.

## Declaration of competing interest

The authors declare that they have no conflicts of interest in this work.

## Appendix A Nomenclature

*A*: cross-sectional area, [m^2^]
*A*_s_: specific surface area, [1/nm]
*C*: concentration, [mol/m^3^]
*C*_df_: diffusion-flow ratio
*d*_m_: molecular diameter of gas, [m]
*d*_p_: local pore diameter, [m]
*d*_p,avg_: average pore diameter, [m]
*d*_pb,avg_: average diameters of the pore body, [m]
*d*_pt_: diameter of the pore throat, [m]
*D_α_*: local diffusivity, [m^2^/s]
*D*_app_: apparent diffusivity, [m^2^/s]
*D*_eff_: normalized effective diffusivity, [m^2^/s]
*D*_Kn_: Knudsen diffusivity, [m^2^/s]
*D*_M_: molecular diffusivity, [m^2^/s]
*D**: normalized diffusivity ($0\leq D^{*}\leq 1$)
*H*: height of the slit, [m]
**J**: mass flux vector, $J=(J_{x},J_{y},J_{z})$, [mol/(m^2^·s)]
*k*_app_: apparent permeability, [m^2^]
*Kn*: Knudsen number
*Kn*_avg_: average Knudsen number
*L_x_*: domain length in *x* direction, [m]
*L_y_*: domain length in *y* direction, [m]
*L_z_*: domain length in *z* direction, [m]
*m*: molecular mass, [kg]
*M*: molar mass, [kg/mol]
*M*_diff_: mass flow rate ratio of the mass diffusion, [kg/s]
*M*_flow_: mass flow rate ratio of the viscous flow, [kg/s]
*n*: number density of gas molecules, [1/m^3^]
*N_x_*: grid number in the *x*direction
*N_y_*: grid number in the *y*direction
*N_z_*: grid number in the *z*direction
*p*: pressure, [Pa]
*R*: radius of the tube, [m]
*R*_ig_: ideal gas constant, *R*_ig_= 8.314 J/(mol·K)
*t*: time, [s]
*T*: temperature, [K]
**u**: velocity vector, $u=(u_{x},u_{y},u_{z})$, [m/s]
**u**_s_: slip velocity on the pore surface, $u_{s}=\left(u_{s,x},u_{s,y},u_{s,z}\right)$, [m/s]
$\bar{v}$: mean molecular velocity, [m/s]
*Z*: compressibility factor

**Greek symbols**
α: empirical parameter
*ε*: porosity
$\lambda_{0}$: unbound mean free path, [m]
$\mu_{0}$: gas bulk dynamic viscosity, [Pa·s]
ρ: gas density, [kg/m^3^]

**Subscripts**
app: apparent value
avg: average value
in: at the inlet
out: at the outlet
